# Human Factors in AI-Driven Digital Solutions for Increasing Physical Activity: Scoping Review

**DOI:** 10.2196/55964

**Published:** 2024-07-03

**Authors:** Elia Gabarron, Dillys Larbi, Octavio Rivera-Romero, Kerstin Denecke

**Affiliations:** 1 Department of Education, ICT and Learning Østfold University College Halden Norway; 2 Norwegian Centre for eHealth Research University Hospital of North Norway Tromsø Norway; 3 Department of Clinical Medicine The University of Tromsø-The Arctic University of Norway Tromsø Norway; 4 Department of Electronic Technology Universidad de Sevilla Sevilla Spain; 5 AI for Health, Institute Patient-centered Digital Health, Department of Engineering and Computer Science Bern University of Applied Sciences Bern Switzerland

**Keywords:** machine learning, ML, artificial intelligence, AI, algorithm, algorithms, predictive model, predictive models, predictive analytics, predictive system, practical model, practical models, deep learning, human factors, physical activity, physical exercise, healthy living, active lifestyle, exercise, physically active, digital health, mHealth, mobile health, app, apps, application, applications, digital health, digital technology, digital intervention, digital interventions, smartphone, smartphones, PRISMA

## Abstract

**Background:**

Artificial intelligence (AI) has the potential to enhance physical activity (PA) interventions. However, human factors (HFs) play a pivotal role in the successful integration of AI into mobile health (mHealth) solutions for promoting PA. Understanding and optimizing the interaction between individuals and AI-driven mHealth apps is essential for achieving the desired outcomes.

**Objective:**

This study aims to review and describe the current evidence on the HFs in AI-driven digital solutions for increasing PA.

**Methods:**

We conducted a scoping review by searching for publications containing terms related to PA, HFs, and AI in the titles and abstracts across 3 databases—PubMed, Embase, and IEEE Xplore—and Google Scholar. Studies were included if they were primary studies describing an AI-based solution aimed at increasing PA, and results from testing the solution were reported. Studies that did not meet these criteria were excluded. Additionally, we searched the references in the included articles for relevant research. The following data were extracted from included studies and incorporated into a qualitative synthesis: bibliographic information, study characteristics, population, intervention, comparison, outcomes, and AI-related information. The certainty of the evidence in the included studies was evaluated using GRADE (Grading of Recommendations Assessment, Development, and Evaluation).

**Results:**

A total of 15 studies published between 2015 and 2023 involving 899 participants aged approximately between 19 and 84 years, 60.7% (546/899) of whom were female participants, were included in this review. The interventions lasted between 2 and 26 weeks in the included studies. Recommender systems were the most commonly used AI technology in digital solutions for PA (10/15 studies), followed by conversational agents (4/15 studies). User acceptability and satisfaction were the HFs most frequently evaluated (5/15 studies each), followed by usability (4/15 studies). Regarding automated data collection for personalization and recommendation, most systems involved fitness trackers (5/15 studies). The certainty of the evidence analysis indicates moderate certainty of the effectiveness of AI-driven digital technologies in increasing PA (eg, number of steps, distance walked, or time spent on PA). Furthermore, AI-driven technology, particularly recommender systems, seems to positively influence changes in PA behavior, although with very low certainty evidence.

**Conclusions:**

Current research highlights the potential of AI-driven technologies to enhance PA, though the evidence remains limited. Longer-term studies are necessary to assess the sustained impact of AI-driven technologies on behavior change and habit formation. While AI-driven digital solutions for PA hold significant promise, further exploration into optimizing AI’s impact on PA and effectively integrating AI and HFs is crucial for broader benefits. Thus, the implications for innovation management involve conducting long-term studies, prioritizing diversity, ensuring research quality, focusing on user experience, and understanding the evolving role of AI in PA promotion.

## Introduction

Physical activity (PA) has been recognized as a cornerstone of a healthy lifestyle since it has demonstrated numerous benefits for both physical and mental well-being [1,2]. Engaging in regular PA has been associated with preventing and managing a range of health conditions, including obesity, diabetes, cardiovascular disease, and multiple sclerosis [2,3]. However, the global population’s engagement in regular PA is often low, with many individuals failing to meet the recommendations necessary for health benefits. This persistent challenge necessitates innovative approaches to motivate and facilitate increased PA participation, and mobile health (mHealth) technologies have emerged as a promising avenue for intervention [4].

The availability of mobile devices and the increasing mobile penetration provide an unprecedented opportunity to leverage mHealth solutions to promote PA [5,6]. Mobile technologies offer persuasive and ubiquitous systems. Equipped with built-in sensors that can monitor and encourage PA in real time, they can facilitate sending personalized reminders and motivational messages [7-10], which have been proven to significantly increase PA [10-12]. However, the effectiveness of mHealth interventions in promoting PA has been limited by the challenge of sustaining engagement over the medium and long term. Mönninghoff et al [13] found that mHealth “can foster small to moderate increases in PA,” and the effects are even maintained long-term, but “the effect size decreases over time.” This is where the integration of artificial intelligence (AI) holds immense promise. AI technology has the potential to deliver effective interventions to promote PA [11,14].

AI can enrich mHealth solutions by offering personalized, adaptive, and tailored interventions that cater to individual preferences and needs. For example, an optimal exercise plan for an individual can be suggested by AI algorithms to help maximize the long-term health utility of the user [15]. This level of customization has the potential to enhance user experience (UX), which in turn could result in increased motivation to engage in PA. Motivation is a critical factor in driving behavior change, especially when adopting and maintaining a physically active lifestyle. AI can also gamify fitness by setting challenges, goals, and rewards, motivating users to increase PA through points, competition, and achievements [16]. Moreover, research indicates that the human-likeness of conversational agents increases adherence to chatbots [17] and compliance with their recommendations [18].

In this context, human factors (HFs) play a pivotal role in the successful integration of AI into mHealth solutions aimed at promoting PA. Understanding and optimizing the interaction between individuals and AI-driven mHealth apps is essential for achieving the desired outcomes [19]. HFs, in the context of AI, involve considerations related to human cognition, behavior, and ergonomics, which are crucial for designing effective and user-friendly mHealth interventions. Bergevi et al [20] explored users’ perceptions of acceptability, engagement, and usability of mHealth solutions that promote PA, healthy diets, or both. They concluded that mHealth services targeting increased PA “should be personalized, dynamic, easily manageable, and reliable.” This study is distinguished from their work by focusing on AI-driven digital solutions.

This research underscores the critical role of PA in promoting overall health and well-being while highlighting the persistent challenge of low engagement in regular PA globally. It emphasizes the potential of mHealth technologies, augmented by AI, to effectively motivate and facilitate increased PA participation. By leveraging AI, mHealth solutions can offer personalized, adaptive interventions tailored to individual preferences and needs, thereby enhancing the UX and motivation. However, the successful integration of AI into mHealth solutions relies on understanding and optimizing HFs, encompassing cognition, behavior, and ergonomics, to ensure effective and user-friendly interventions. Specifically, this study aims to address the following research question, what are the key HFs influencing the effectiveness and adoption of AI-driven digital solutions aimed at promoting PA? Our objective is to review and describe the current evidence on the HFs in AI-driven digital solutions for increasing PA.

## Methods

### Overview

We have conducted a scoping review to capture current evidence on HFs in AI-driven digital solutions for increasing PA. A scoping review is a systematic approach used to map and synthesize existing literature on a broad topic, providing an overview of key concepts, sources, and knowledge gaps. Our review followed the PRISMA-ScR (Preferred Reporting Items for Systematic Reviews and Meta-Analyses extension for Scoping Reviews) [21].

### Search Strategy

We have searched for publications including keywords related to PA (ie, “physical activity;” “exercise;” “active lifestyle;” “sedentary behaviour;” “inactivity;” “resistance training;” “exergaming;” “walking;” “swimming;” “jogging;” “climbing”), artificial intelligence (ie, “artificial intelligence;” “AI;” “machine learning;” “deep learning;” “natural language processing;” “neural networks;” “sentiment analysis”), and human factors (ie, “usability;” “task performance;” “satisfaction;” “workload;” “human errors;” “user perception;” “cognitive factors;” “mental model;” “context awareness;” “automation bias;” “teamworking;” “user experience;” “acceptance;” “acceptability;” “task analysis;” “handover;” “patient interaction;” “human factors;” “ergonomics”) in their titles and abstracts. No language or year limitations were used. The full search strategy can be found in Multimedia Appendix 1.

The data search was performed on August 29, 2023. The database search was done by a single author (EG) and covered PubMed, Embase, and IEEE Xplore. Another author (DL) carried out a search on Google Scholar and selected the first 100 entries. Finally, DL used a snowballing approach to identify additional relevant studies cited in only the included publications.

### Eligibility and Selection Process

Inclusion and exclusion criteria are presented in Textbox 1.

**Inclusion and exclusion criteria.**

**Inclusion criteria**

- Primary studies that described an artificial intelligence (AI)–based digital solution
- AI-based digital solutions aimed at increasing physical activity
- Publications that reported results from testing the AI-based solution related to physical activity behavior

**Exclusion criteria**

- Publications that did not meet all 3 inclusion criteria

All references were uploaded to EndNote (version 20.6; Clarivate) [22] and Rayyan (Qatar Computing Research Institute) [23]. After duplicates were removed, 2 authors (EG and DL) independently assessed the eligibility of the remaining publications by checking their titles and abstracts. Two additional authors (KD and OR-R) checked the full text of the eligible papers after the title and abstract screening. After the full-text screening, the selected papers were included in a qualitative synthesis.

### Data Items and Data Extraction

Two authors (KD and OR-R) extracted the following data: bibliographic information (publication year and country); study characteristics (study design, type of evaluation, research methods, primary and secondary measures, materials, and theoretical foundations); population (number of participants, age, and gender); intervention (intervention design, duration, and follow-ups); comparison (control group or groups and pre-post evaluation or other); outcomes (primary and secondary outcomes); and AI-related information (technology type, main purpose, platform, and HFs).

OR-R identified and assigned codes representative of the main purpose of the AI model implemented in each of the systems studied. The 3 main purposes of the AI models implemented in the studied systems were identified as personalization, communication, and human activity recognition. Personalization includes all AI models analyzed whose main purpose was to adapt the digital solution or intervention to the patient’s needs, conditions, and preferences. The second group includes models that enabled a communication pathway with patients. Finally, human activity recognition includes all models that enable the detection of user behaviors, particularly PA. OR-R and KD reviewed the assigned codes and created a classification of these by consensus.

### Certainty of the Evidence

The certainty of the evidence on the outcomes was assessed by a single author (EG) by drawing on the GRADE (Grading of Recommendations Assessment, Development, and Evaluation) criteria [24] and verified by the rest of the coauthors.

## Results

### Study Selection

**Figure 1:**
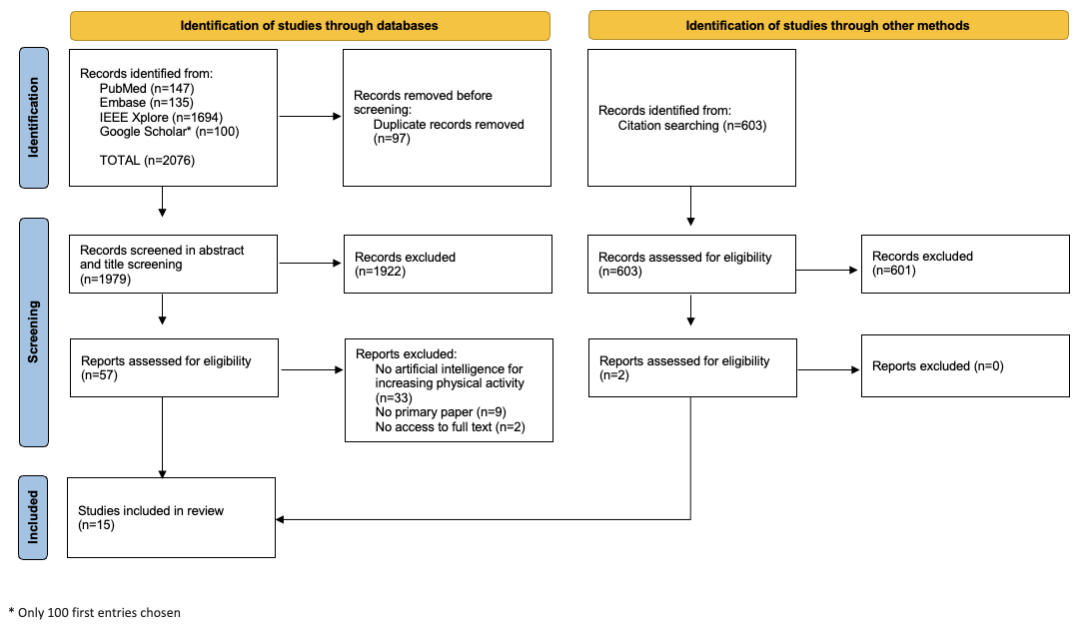
Flowchart diagram of the selection process.

A total of 2076 articles were identified in the data search. After removing duplicates, 1979 titles and abstracts were screened for eligibility. Of those, 13 publications met the inclusion criteria [25-37]. The snowballing approach identified 2 additional publications [38,39]; therefore, the final number of publications included in this review was 15 (Figure 1 shows the PRISMA [Preferred Reporting Items for Systematic Reviews and Meta-Analyses] flowchart).

The list of publications excluded during the full-text search and the reasons for their exclusion are reported in Multimedia Appendix 2.

### Description of the Included Publications

The 15 included articles were published between 2015 and 2023. Countries of origin of these studies were: United States (n=3) [25,26,36], Australia (n=2) [32,38], South Korea (n=2) [29,35], the Netherlands (n=1) [31], Italy (n=1) [27], Belgium and Italy (n=1) [30], Thailand (n=1) [37], and Taiwan (n=1) [33]. A total of 3 publications did not specify in which country the study was performed [28,34,39].

Regarding the study design, 8 publications followed a quasi-experimental approach [26-29,32,35,38,39], 5 were randomized controlled trials [25,30,33,36,37], and 2 were exploratory studies [31,34].

Only 4 of the 15 included publications explicitly mentioned their theoretical foundations. The following theoretical approaches were cited in these 4 publications: the Fogg Model for behavior change [25,31], Capability, Opportunity, and Motivation model of Behavior [32]; learning theory [25], social cognitive theory [25], and the Transtheoretical Model [39].

The main technical characteristics of the 15 included publications are presented in Table 1.

**Table 1:** Main technical characteristics of the included artificial intelligence (AI)–based solutions.

Author and year	AI tech type	AI purpose	AI techniques	System platform	Human factors
Rabbi et al (2015) [25]	RS^a^	Personalization	Gaussian mixture model	MyBehavior (Mobile app)	User experience
Rabbi et al (2018) [26]	RS	Personalization and HAR^b^	Data clustering algorithm and sequential decision-making algorithm (multiarmed bandit)	MyBehaviorCBP (Mobile app)	Acceptability
Fadhil et al (2019) [27]	CA^c^	Communication	Fine state machine and multi class support vector machine	Chatbot stand-alone	Acceptability
Davis et al (2020) [28]	CA	Communication	Unknown	IBM Watson digital assistant AI software running on Slack	User experience
Luštrek et al (2021) [30]	RS	Personalization and HAR	Random forest	App with wristband	Acceptability, attitude, and performance expectancy
Joo et al (2021) [29]	RS+HAR	Personalization and HAR	Feature point extraction and part affinity fields (machine learning technology with top-down segmentation)	Weelo (web-based fitness program)	Satisfaction, usability, and usefulness
Pelle et al (2021) [31]	RS	Personalization and proposes challenging, achievable, and tailored goals	Machine learning compromising a dynamic model (contextual multiarmed bandit approach)	A stand-alone mobile health app	Usability
To et al (2021) [32]	CA	Personalization and communication	Unknown	DialogFlow (Google), Fitbit Flex, and messenger app	Usability and acceptability
Lin et al (2022) [33]	RS	Personalization and provides a personal training program	Decision tree	AIoT^d^, mobile app, and web application	Usability
Park et al (2022) [34]	HAR	HAR	Convolutional neuronal networks	Mobile app	Satisfaction, acceptability, and task performance
Seok et al (2022) [35]	RS	Communication	Large-scale modular behavior networks with inferred contexts and probabilistic model and Russel’s arousal-variance model	TouchCare system: wearable watch, touchpad sensors, TouchCare app, and context-aware AI	Satisfaction
Bates et al (2023) [36]	RS	Personalization and real-time feedback on form and resistance for each task in the training program	Unknown	Tonal AI (commercially available product)	Satisfaction
Thiengwittayaporn et al (2023) [37]	RS	Personalization and patient disease stage	Decision tree classification	Mobile app	Satisfaction
Maher et al (2020) [38]	CA	Communication and personalization	Unknown	IBM Watson	Acceptability

^a^RS: recommender system.

^b^HAR: human activity recognition.

^c^CA: conversational agent.

^d^AIoT: artificial intelligence of things.

### AI-Driven Technology and HFs

The most common AI technology type was recommender systems, described in 10 of the 15 included publications [25,26,29-31,33,35-37,39]. In addition to the recommender system, one of these publications also included computer vision [29]. Conversational agents were the second most used AI technology, as described in 4 publications [27,28,32,38]. One of them was integrated into a social media platform, namely Slack [28]. One study tested human activity recognition [34]. Details of the AI technology, systems, or platforms used in the included studies are summarized in Table 1.

Regarding the considered HFs, the most commonly evaluated were acceptability [26,32,34,38,39] and satisfaction [29,34-37], both reported in 5 publications. Usability was the next most considered and evaluated HF, as reported in 4 papers [29,31-33]. Usefulness was assessed in 2 publications [26,29]. Other considered and evaluated HFs were engagement [38], UX or individual perception [25], and task performance [34].

The studies that resulted in increased PA and had a moderate certainty of evidence were chatbot systems with integrated recommender systems. Although the usability of some of those systems was considered poor [32], they were perceived positively. Several papers gained interesting results regarding HFs. Given the variety of systems, a generalization for all 15 studies is difficult.

The automated collection of data needed for personalization and recommendation is an important aspect. In total, 5 systems involved fitness trackers [26,30,32,35,38] to enable automated data collection. They can be grouped into mobile-based activity tracking using movement sensors in the phone [25], dedicated fitness trackers [30,32,38], specifically an accelerometer in the wristband [30], Fitbit Flex 1 activity tracker (Fitbit LLC) [32], and Garmin Vivofit4 tracker (Garmin) [38], and smartwatches [35]. Rabbi et al [25] concluded that automated data collection would be useful. The studies involving chatbots concluded that users have high expectations regarding the chatbot’s knowledge and capabilities [28]. Human likeness is reported as a success factor of such systems. Relevant aspects leading to the efficacy of the system include the human-like qualities of the chatbot and the personalization of the suggestions [28,32,39], that is, chatbots or digital assistants should have a personality, have humor, be able to act with spontaneous behavior, and in a diverse, nonrepetitive manner [28,32]. They should provide the correct answers. For successful recommendations, it is essential to learn the personal preferences of users so that suggestions can be made that fit into personal routines and lifestyles [39].

Even a combination of human agents and digital agents was reported to be better accepted than pure virtual support [27]. Beyond that, access to a system anywhere and anytime is well perceived [31]—and this is reflected by the fact that most systems included in this study are delivered as mobile apps (instead of desktop apps). Exercises and recommendations are successful in this setting when they can be easily integrated into the daily lives of the users [31].

### Population, Interventions, and Comparison

A total of 899 individuals participated in the included publications. Of those, 60.7% (546) were female participants. The reported average ages of these participants ranged from 18.7 to 84.4 years. In total, 6 out of the 15 studies tested their solutions on participants with mean ages of around 50 years or older [28,31-33,37,38], while 6 studies predominantly included participants with a mean age of 40 years or younger [25,27,29,34,36,39]. Two studies did not specify the gender or age of participants [30,35].

The intervention of the included studies lasted between 2 and 26 weeks.

Prepost evaluations were carried out in 6 of the publications to evaluate the impact of the AI-driven intervention [26,29,32,35,38,39]. In 4 publications, control groups were used to assess the impact [25,30,34,36]. In 5 of the publications, the comparison methods used to assess the impact of the AI-driven intervention on increasing PA were not clearly reported [27,28,31,33,37].

### Outcomes and Certainty of the Evidence

The effectiveness of AI-driven technologies for increasing PA was shown in 5 publications [28,32,36,38,39]. Three of these publications tested conversational agents [28,32,38], while the other 2 focused on recommender systems [36,39]. The analysis, based on GRADE guidelines, found moderate certainty in the evidence supporting this statement. Further details about the proven effect of these studies and the certainty of the evidence on these findings are reported in Table 2.

**Table 2:** Certainty of the evidence (artificial intelligence for increasing physical activity [PA]).

Outcome	Effect	Participants (studies)	Certainty of the evidence (GRADE^a^)^b^	Comment
Increased number of steps, distance walked, or time spent on PA Follow-up: mean 9.4 weeks	Increased walked distance [36] exceeded step goal [28] more steps [32] increased walking minutes [39] increased time spent on PA [38]	n=260 (3 pre-post studies, 1 RCT^c^, and 1 observational study)	B: moderate	We have a moderate level of confidence that the actual impact closely aligns with the estimated effect.
Change in PA behavior and abilities to perform behavior Follow-up: mean 13.3 weeks	Feeling more stimulated to engage in PAs [30] change in walking behaviors [25] improved behaviors related to PA [35] improved ability to do sports [37]	n=98 reported (number not explicitly reported in 2 studies) 3 RCTs, 1 pre-post study	D: very low	We have a very low level of confidence in the estimated effect.

^a^GRADE: Grading of Recommendations Assessment, Development, and Evaluation.

^b^Scale of 4 degrees, where A denotes the highest quality and D denotes the lowest quality.

^c^RCT: randomized controlled trial.

In total, 4 of the included articles also showed that AI technologies have an effect on changing PA behavior (ie, feeling more stimulated to engage in PAs, change in walking behavior, improved behavior related to PA, or improved ability to perform PA) [25,30,35,37]. All these publications were recommender systems [25,30,35,37] and found a positive effect of AI-driven technology on changing PA behavior. However, the analysis, based on GRADE guidelines, found very low certainty evidence supporting this statement.

## Discussion

### Principal Results

In this scoping review, we aimed to identify and describe the current evidence on HFs in AI-driven digital solutions for increasing PA. The results showed that the most common AI technology used in digital solutions for PA was recommender systems, followed by conversational agents. User acceptability and satisfaction were the most commonly evaluated HFs in the included studies. Some studies also evaluated the usability of AI-driven digital solutions for PA.

We have identified studies that provide evidence that AI-driven digital technologies have the potential to increase PA (eg, number of steps, distance walked, or time spent on PA). Furthermore, AI-driven technology, particularly recommender systems and chatbots, seems to have the potential to influence changes in PA behavior. Although these studies offer valuable insights by demonstrating positive outcomes through various AI-driven technologies for enhancing PA, the evidence is still very limited. The main findings are presented in Table 3.

**Table 3:** Summary of main findings.

Included in review	Findings (N=15 studies; covering a total of 899 study participants)
Interventions duration	Interventions lasted between 2 and 26 weeks
Used AI^a^ technologies	Recommender systems (described in 10/15 studies) Conversational agents (described in 4/15 studies) Human activity recognition (described in 1 study)
Human factors	Acceptability (evaluated in 5/15 studies) Satisfaction (evaluated in 5/15 studies) Usability (evaluated in 4/15 studies) Usefulness (evaluated in 2/15 studies) Engagement (evaluated in 1 study) User experience (evaluated in 1 study) Task performance (evaluated in 1 study)
Effectiveness of AI-driven technologies for increasing PA^b^	Moderate evidence: AI-driven digital technologies have the potential to increase PA (eg, number of steps, distance walked, or time spent on PA) Very low evidence: Recommender systems and chatbots, seems to have the potential to influence changes in PA behavior

^a^AI: artificial intelligence.

^b^PA: physical activity.

### Comparison With Previous Work

In the included studies, we recognized several benefits of AI integrated into digital solutions for increasing PA, such as the ability to adapt the solution to the patient’s physical capacity, current activity, and psychological profile [8,11,30]. AI can monitor activity and inactivity and predict bodily occurrences, which is especially relevant for older people [40]. AI can also simulate the role of a personal trainer, provide guidance, form correction, and motivation [37] through voice- or text-based interactions. Users can receive real-time feedback and support during their workouts [8,10] which would be difficult to achieve with non-AI digital solutions. AI algorithms can analyze user data such as fitness levels, health conditions, and preferences and provide personalized exercise recommendations [11]. The activities or other suggestions are tailored to the specific needs and goals of the user, increasing the likelihood of adherence. Real-time feedback can be shared with the user. Previous studies found that activity tracking combined with real-time, personalized text messages can significantly increase PA and further affirm text messaging as an effective health behavior modifier [10-12]. However, in our review, researchers concluded that their solution did not achieve sufficient adherence to the exercise program [28,30]. The entire potential of personalization techniques has not yet been implemented in the solutions, as Luštrek et al [30] concluded that personalization, simplicity, ease of use, and avoiding information overload could be improved.

AI algorithms can continuously learn from user interactions and feedback to refine and improve the UX. This iterative process leads to more effective and engaging solutions over time. For example, the continuous interaction that chatbots can provide was reported to be useful in helping users increase regular PA and in helping them stay motivated to participate in PA [32]. Studies have already found that the human-likeness or anthropomorphisms of a chatbot increase the likelihood that users comply with the chatbot’s recommendations [18]. Roy and Naidoo [17] found that human qualities like warmth and competence are contributing to a positive UX and possibly to an increased adherence to the digital solution [17].

We only found 5 studies involving sensors to measure PA [26,30,32,35,38]. Dedicated fitness trackers seem to be more prominent to be involved in solutions increasing PA. Mobile-based activity tracking and smartwatches were only implemented in one solution. A reason might be that users prefer to use systems they already use; that is, integration with existing tools like fitness trackers is desired by users, as found by Wang et al [41]. The landscape of wearables and sensors that could be used for PA tracking is much larger than was found in our research [42]. The integration of sensors with AI could help analyze the data streams and promote an increase in PA [43]. Additionally, it could assist in monitoring PA among individuals affected by health conditions [44-46]. We hypothesize that existing research focuses on sensors that are well-known, not very intrusive, and therefore probably more accepted by users of solutions for increasing PA.

AI can gamify the fitness experience by setting challenges, goals, and rewards. Users are motivated to increase PA by earning points, competing with friends, or unlocking achievements. Xu et al [16] found in their review that gamification interventions could increase PA participation. Interestingly, none of our included studies explicitly reported about gamification elements.

### Do We Have Enough Evidence on AI’s Effectiveness in Increasing PA?

In total, 5 of the included studies provide moderate evidence of AI’s effectiveness for increasing PA [28,32,36,38,39]. However, these studies involve short interventions lasting from 6 to 12 weeks. Hence, the significant effect might be influenced by this brief follow-up period, similar to other mHealth interventions [13]. The estimated time needed to form habits of complex behaviors such as exercise behavior is 12 weeks [47]. Thus, longer intervention studies are needed to assess the potential long-term effectiveness of AI-driven technologies for increasing PA.

Out of the 260 participants in these 5 studies [28,32,36,38,39], 72.3% (188) of them were women, and the majority were aged between 40 and 50 years. Further studies are needed to investigate the effects of these AI-driven technologies on participants with different sociodemographic characteristics, as well as those with health conditions for which exercise aids in managing the disease and preventing complications [1,2,4].

There is very limited and low-quality evidence supporting the impact of AI-driven technologies on changing PA behavior and the ability to perform such behavior [25,30,35,37]. In these cases, the durations of the interventions varied, ranging from as short as 3 to 4 weeks [25,37], to as long as 20 to 26 weeks [30,35]. Similar to previous cases, the majority of participants in these 4 studies were women, comprising 83.7% (82/98) reported participants. While research indicates that gender is one of the factors influencing the use of health-related technologies [48,49], technologies aimed at increasing PA should be tested, personalized, and accessible for all demographic groups.

### What Is the Role of HFs on the AI-Based PA Solutions?

Most of the included AI-based PA systems showed positive results in terms of HFs related to their use. However, no study aimed to evaluate how the AI component could independently influence HFs such as user acceptance, perceived ease of use, or perceived usefulness. Many studies used AI techniques to personalize the PA system based on the authors’ assumptions about the persuasive power of personalization that could lead to greater motivation and thus result in greater intention to use, adoption, and engagement. However, no study has tested these hypotheses. In this regard, more research is still needed to identify the role of AI components in HFs affecting PA systems. In addition, no study has focused on whether the inclusion of AI could lead to a change in the role of HFs, as has been the case with traditional technologies.

### Limitations

There were some identified limitations in this scoping review. Even though we did not have a language limitation in the search strategy, all the included studies were in English. Therefore, we could have missed relevant AI-driven solutions published in other languages. The included studies were mainly from diverse high-income countries, restricting generalization to low- and middle-income countries. In addition, the studies included in the scoping review had an intervention period of a maximum of 26 weeks, showing only the short-term effect of the AI-driven solutions. All studies were included in the review, irrespective of the assessed quality of the evidence. However, the results of the included studies were reported separately according to the quality of the evidence, minimizing misinterpretation of the data.

### Conclusions

This study synthesized current evidence on the effectiveness and potential of AI-driven digital solutions for increasing PA. Although the included studies offer valuable insights by demonstrating positive outcomes through various AI-driven technologies for enhancing PA, the evidence is still very limited. While some studies demonstrated moderate evidence of AI’s effectiveness in increasing PA, these interventions were typically short-term. Longer-term studies are necessary to assess the sustained impact of AI-driven technologies on behavior change and habit formation. Additionally, further research is needed to investigate the effects of AI-driven interventions on diverse populations, including individuals with varying sociodemographic characteristics and conditions. Moreover, the evidence regarding the impact of AI-driven technologies on changing PA behavior remains limited and of low quality. There is a need for rigorous studies to evaluate the effectiveness of these interventions, particularly in terms of their ability to induce long-term behavior change. Furthermore, while most AI-based PA systems demonstrated positive results in terms of UX, there is a lack of research focusing on the independent influence of AI components on HFs, such as user acceptance and perceived usefulness. Additionally, more investigation is required to understand how the inclusion of AI may alter the role of HFs in PA systems compared to traditional technologies.

In conclusion, while AI-driven digital solutions hold significant promise for promoting PA and improving public health outcomes, addressing these limitations and challenges will be crucial for maximizing their effectiveness and accessibility. Continued research efforts in these areas are essential for advancing our understanding of the role of AI in PA promotion and ensuring the development of evidence-based interventions that benefit diverse populations.

## Appendix

Multimedia Appendix 1 Full search strategy.

Multimedia Appendix 2 Excluded papers in full text eligibility phase.

Multimedia Appendix 3 PRISMA checklist.

## Abbreviations

AI: artificial intelligence
GRADE: Grading of Recommendations Assessment, Development and Evaluation
HF: human factor
mHealth: mobile health
PA: physical activity
PRISMA: Preferred Reporting Items for Systematic Reviews and Meta-Analyses
PRISMA-ScR: Preferred Reporting Items for Systematic Reviews and Meta-Analyses extension for Scoping Reviews
UX: user experience

## References

[1] Schuch FB, Vancampfort D (2021). Physical activity, exercise, and mental disorders: it is time to move on. Trends Psychiatry Psychother.

[2] (2018). Global action plan on physical activity 2018-2030: more active people for a healthier world. World Health Organization.

[3] Rhodes RE, Janssen I, Bredin SS, Warburton DE, Bauman A (2017). Physical activity: health impact, prevalence, correlates and interventions. Psychol Health.

[4] McGarrigle L, Todd C (2020). Promotion of physical activity in older people using mHealth and eHealth technologies: rapid review of reviews. J Med Internet Res.

[5] McIntosh J, Jay S, Hadden N, Whittaker P (2017). Do E-health interventions improve physical activity in young people: a systematic review. Public Health.

[6] Bickmore TW, Schulman D, Sidner C (2013). Automated interventions for multiple health behaviors using conversational agents. Patient Educ Couns.

[7] Laranjo L, Ding D, Heleno B, Kocaballi B, Quiroz JC, Tong HL, Chahwan B, Neves AL, Gabarron E, Dao KP, Rodrigues D, Neves GC, Antunes ML, Coiera E, Bates DW (2021). Do smartphone applications and activity trackers increase physical activity in adults? Systematic review, meta-analysis and metaregression. Br J Sports Med.

[8] An R, Shen J, Wang J, Yang Y (2024). A scoping review of methodologies for applying artificial intelligence to physical activity interventions. J Sport Health Sci.

[9] Catellani P, Biella M, Carfora V, Nardone A, Brischigiaro L, Manera MR, Piastra M (2023). A theory-based and data-driven approach to promoting physical activity through message-based interventions. Front Psychol.

[10] Vandelanotte C, Trost S, Hodgetts D, Imam T, Rashid M, To QG, Maher C (2023). Increasing physical activity using an just-in-time adaptive digital assistant supported by machine learning: a novel approach for hyper-personalised mHealth interventions. J Biomed Inform.

[11] Hassoon A, Baig Y, Naiman DQ, Celentano DD, Lansey D, Stearns V, Coresh J, Schrack J, Martin SS, Yeh H, Zeilberger H, Appel LJ (2021). Randomized trial of two artificial intelligence coaching interventions to increase physical activity in cancer survivors. NPJ Digit Med.

[12] Martin SS, Feldman DI, Blumenthal RS, Jones SR, Post WS, McKibben RA, Michos ED, Ndumele CE, Ratchford EV, Coresh J, Blaha MJ (2015). mActive: A randomized clinical trial of an automated mHealth intervention for physical activity promotion. J Am Heart Assoc.

[13] Mönninghoff A, Kramer JN, Hess AJ, Ismailova K, Teepe GW, Tudor Car L, Müller-Riemenschneider F, Kowatsch T (2021). Long-term effectiveness of mHealth physical activity interventions: systematic review and meta-analysis of randomized controlled trials. J Med Internet Res.

[14] Oh YJ, Zhang J, Fang M, Fukuoka Y (2021). A systematic review of artificial intelligence chatbots for promoting physical activity, healthy diet, and weight loss. Int J Behav Nutr Phys Act.

[15] Fang J, Lee VCS, Ji H, Wang H (2022). Enhancing digital health services: a machine learning approach to personalized exercise goal setting. ArXiv, arXiv:2204.00961.

[16] Xu L, Shi H, Shen M, Ni Y, Zhang X, Pang Y, Yu T, Lian X, Yu T, Yang X, Li F (2022). The effects of mHealth-based gamification interventions on participation in physical activity: systematic review. JMIR Mhealth Uhealth.

[17] Roy R, Naidoo V (2021). Enhancing chatbot effectiveness: the role of anthropomorphic conversational styles and time orientation. Journal of Business Research.

[18] Adam M, Wessel M, Benlian A (2020). AI-based chatbots in customer service and their effects on user compliance. Electron Markets.

[19] Kushniruk AW, Borycki EM (2023). Human factors in healthcare IT: management considerations and trends. Healthc Manage Forum.

[20] Bergevi J, Andermo S, Woldamanuel Y, Johansson U, Hagströmer M, Rossen J (2022). User perceptions of eHealth and mHealth services promoting physical activity and healthy diets: systematic review. JMIR Hum Factors.

[21] Tricco AC, Lillie E, Zarin W, O'Brien KK, Colquhoun H, Levac D, Moher D, Peters MD, Horsley T, Weeks L, Hempel S, Akl EA, Chang C, McGowan J, Stewart L, Hartling L, Aldcroft A, Wilson MG, Garritty C, Lewin S, Godfrey CM, Macdonald MT, Langlois EV, Soares-Weiser K, Moriarty J, Clifford T, Tunçalp Ö, Straus SE (2018). PRISMA extension for scoping reviews (PRISMA-ScR): checklist and explanation. Ann Intern Med.

[22] EndNote 2024. Clarivate.

[23] Rayyan 2022. Rayyan.

[24] Murad MH, Mustafa RA, Schünemann HJ, Sultan S, Santesso N (2017). Rating the certainty in evidence in the absence of a single estimate of effect. Evid Based Med.

[25] Rabbi M, Pfammatter A, Zhang M, Spring B, Choudhury T (2015). Automated personalized feedback for physical activity and dietary behavior change with mobile phones: a randomized controlled trial on adults. JMIR Mhealth Uhealth.

[26] Rabbi M, Aung MS, Gay G, Reid MC, Choudhury T (2018). Feasibility and acceptability of mobile phone-based auto-personalized physical activity recommendations for chronic pain self-management: pilot study on adults. J Med Internet Res.

[27] Fadhil A, Wang Y, Reiterer H (2019). Assistive conversational agent for health coaching: a validation study. Methods Inf Med.

[28] Davis CR, Murphy KJ, Curtis RG, Maher CA (2020). A process evaluation examining the performance, adherence, and acceptability of a physical activity and diet artificial intelligence virtual health assistant. Int J Environ Res Public Health.

[29] Joo S, Lee C, Joo N, Kim C (2021). Feasibility and effectiveness of a motion tracking-based online fitness program for office workers. Healthcare (Basel).

[30] Luštrek M, Bohanec M, Cavero Barca C, Ciancarelli MC, Clays E, Dawodu AA, Derboven J, De Smedt D, Dovgan E, Lampe J, Marino F, Mlakar M, Pioggia G, Puddu PE, Rodríguez JM, Schiariti M, Slapničar G, Slegers K, Tartarisco G, Valič J, Vodopija A (2021). A personal health system for self-management of congestive heart failure (HeartMan): development, technical evaluation, and proof-of-concept randomized controlled trial. JMIR Med Inform.

[31] Pelle T, van der Palen J, de Graaf F, van den Hoogen FHJ, Bevers K, van den Ende CHM (2021). Use and usability of the dr. Bart app and its relation with health care utilisation and clinical outcomes in people with knee and/or hip osteoarthritis. BMC Health Serv Res.

[32] To QG, Green C, Vandelanotte C (2021). Feasibility, usability, and effectiveness of a machine learning-based physical activity chatbot: quasi-experimental study. JMIR Mhealth Uhealth.

[33] Lin CC, Kuo LC, Lin YS, Chang CM, Hu FW, Chen YJ, Lin CT, Su FC (2022). AIoT-based ergometer for physical training in frail elderly with cognitive decline: a pilot randomized control trial. J. Med. Biol. Eng.

[34] Park J, Chung SY, Park JH (2022). Real-time exercise feedback through a convolutional neural network: a machine learning-based motion-detecting mobile exercise coaching application. Yonsei Med J.

[35] Seok JW, Kwon Y, Lee H (2022). Feasibility and efficacy of TouchCare system using application for older adults living alone: a pilot pre-experimental study. BMC Geriatr.

[36] Bates NA, Huffman A, Goodyear E, Nagai T, Rigamonti L, Breuer L, Holmes BD, Schilaty ND (2023). Physical clinical care and artificial-intelligence-guided core resistance training improve endurance and patient-reported outcomes in subjects with lower back pain. Clin Biomech (Bristol, Avon).

[37] Thiengwittayaporn S, Wattanapreechanon P, Sakon P, Peethong A, Ratisoontorn N, Charoenphandhu N, Charoensiriwath S (2023). Development of a mobile application to improve exercise accuracy and quality of life in knee osteoarthritis patients: a randomized controlled trial. Arch Orthop Trauma Surg.

[38] Maher CA, Davis CR, Curtis RG, Short CE, Murphy KJ (2020). A physical activity and diet program delivered by artificially intelligent virtual health coach: proof-of-concept study. JMIR Mhealth Uhealth.

[39] Rabbi M, Aung M, Zhang M, Choudhury T (2015). MyBehavior: automatic personalized health feedback from user behaviors and preferences using smartphones.

[40] Bhatia M (2024). An AI-enabled secure framework for enhanced elder healthcare. Engineering Applications of Artificial Intelligence.

[41] Wang B, Asan O, Zhang Y (2024). Shaping the future of chronic disease management: insights into patient needs for AI-based homecare systems. Int J Med Inform.

[42] Kazanskiy NL, Khonina SN, Butt MA (2024). A review on flexible wearables – recent developments in non-invasive continuous health monitoring. Sensors and Actuators A: Physical.

[43] Wang J, Yang Y, Liu H, Jiang L (2023). Enhancing the college and university physical education teaching and learning experience using virtual reality and particle swarm optimization. Soft Comput.

[44] Gupta N, Kasula V, Sanmugananthan P, Panico N, Dubin AH, Sykes DA, D'Amico RS (2024). SmartWear body sensors for neurological and neurosurgical patients: a review of current and future technologies. World Neurosurg X.

[45] Carter LG, Ford CD (2023). Promoting physical activity in clinical practice through wearable technology. J Am Assoc Nurse Pract.

[46] Coccia M (2020). Deep learning technology for improving cancer care in society: new directions in cancer imaging driven by artificial intelligence. Technology in Society.

[47] Lally P, van Jaarsveld CHM, Potts HWW, Wardle J (2009). How are habits formed: modelling habit formation in the real world. Euro J Social Psych.

[48] Yang Y, Koenigstorfer J (2021). Determinants of fitness app usage and moderating impacts of education-, motivation-, and gamification-related app features on physical activity intentions: cross-sectional survey study. J Med Internet Res.

[49] Lupton D, Maslen S (2019). How women use digital technologies for health: qualitative interview and focus group study. J Med Internet Res.

