# Supplementary material for: Human Factors in AI-Driven Digital Solutions for Increasing Physical Activity: Scoping Review
Source: JMIR Hum Factors. 2024 Jul 3;11:e55964. doi: 10.2196/55964 (PMC11255529; doi:10.2196/55964)
Supplement: Multimedia Appendix 1 [file humanfactors_v11i1e55964_app1.docx]

**Table S1**. Full search strategy.

Search date: 29^th^ August 2023

| **Database** | **Search engine** | **Results** |
| --- | --- | --- |
| **PubMed** | ((((((((((((physical activity[Title/Abstract]) OR (exercise[Title/Abstract])) OR (active lifestyle[Title/Abstract])) OR (sedentary behaviour[Title/Abstract])) OR (inactivity[Title/Abstract])) OR (resistance training[Title/Abstract])) OR (exergaming[Title/Abstract])) OR (walking[Title/Abstract])) OR (swimming[Title/Abstract])) OR (jogging[Title/Abstract])) OR (climbing[Title/Abstract])) AND (((((((artificial intelligence[Title/Abstract]) OR (AI[Title/Abstract])) OR (machine learning[Title/Abstract])) OR (deep learning[Title/Abstract])) OR (natural language processing[Title/Abstract])) OR (neural networks[Title/Abstract])) OR (sentiment analysis[Title/Abstract]))) AND (((((((((((((((((((usability[Title/Abstract]) OR (task performance[Title/Abstract])) OR (satisfaction[Title/Abstract])) OR (workload[Title/Abstract])) OR (human errors[Title/Abstract])) OR (user perception[Title/Abstract])) OR (cognitive factors[Title/Abstract])) OR (mental model[Title/Abstract])) OR (context awareness[Title/Abstract])) OR (automation bias[Title/Abstract])) OR (teamworking[Title/Abstract])) OR (user experience[Title/Abstract])) OR (acceptance[Title/Abstract])) OR (acceptability[Title/Abstract])) OR (task analysis[Title/Abstract])) OR (handover[Title/Abstract])) OR (patient interaction[Title/Abstract])) OR (human factors[Title/Abstract])) OR (ergonomics[Title/Abstract])) | 147 |
| **EMBASE** | **1. (physical activity or exercise or active lifestyle or sedentary behaviour or inactivity or resistance training or exergaming or walking or swimming or jogging or climbing).ab.**  **2. (artificial intelligence or AI or machine learning or deep learning or natural language processing or neural networks or sentiment analysis).ab.**  **3. (usability or task performance or satisfaction or workload or human errors or user perception or cognitive factors or mental model or context awareness or automation bias or teamworking or user experience or acceptance or acceptability or task analysis or handover or patient interaction or human factors or ergonomics).ab.**  **4. 1 and 2**  **5. 3 and 4** | 135 |
| **IEEE Xplore** | ((("Document Title":”physical activity” OR “exercise” OR “active lifestyle” OR “sedentary behaviour” OR “inactivity” OR “resistance training” OR “exergaming” OR “walking” OR “swimming” OR “jogging” OR “climbing”) AND ("Document Title":”artificial intelligence” OR “AI” OR “machine learning” OR “deep learning” OR “natural language processing” OR “neural networks” OR “sentiment analysis”) AND ("Document Title":“usability” OR “task performance” OR “satisfaction” OR “workload” OR “human errors” OR “user perception" OR “cognitive factors” OR “mental model” OR “context awareness” OR “automation bias” OR “teamworking” OR “user experience”OR “acceptance” OR “acceptability” OR “task analysis” OR “handover” OR “patient interaction” OR “human factors” OR “ergonomics”)) OR (("Abstract":”physical activity” OR “exercise” OR “active lifestyle” OR “sedentary behaviour” OR “inactivity” OR “resistance training” OR “exergaming” OR “walking” OR “swimming” OR “jogging” OR “climbing”) AND ("Abstract":”artificial intelligence” OR “AI” OR “machine learning” OR “deep learning” OR “natural language processing” OR “neural networks” OR “sentiment analysis”) AND ("Abstract":“usability” OR “task performance” OR “satisfaction” OR “workload” OR “human errors” OR “user perception" OR “cognitive factors” OR “mental model” OR “context awareness” OR “automation bias” OR “teamworking” OR “user experience”OR “acceptance” OR “acceptability” OR “task analysis” OR “handover” OR “patient interaction” OR “human factors” OR “ergonomics”)))Limit: Conferences and Journals | **1694** |
| **Google Scholar** | (physical activity OR exercise OR active lifestyle OR sedentary behaviour OR inactivity OR resistance training OR exergaming OR walking OR swimming OR jogging OR climbing) AND (artificial intelligence OR AI OR machine learning OR deep learning OR natural language processing OR neural networks OR sentiment analysis) AND (usability OR task performance OR satisfaction OR workload OR human errors OR user perception OR cognitive factors OR mental model OR context awareness OR automation bias OR teamworking OR user experience OR acceptance OR acceptability OR task analysis OR handover OR patient interaction OR human factors OR ergonomics)  ***** Only 100 first entries chosen** | **100** |
|  | **TOTAL** | **2076** |
