# Supplementary material for: Human Factors in AI-Driven Digital Solutions for Increasing Physical Activity: Scoping Review
Source: JMIR Hum Factors. 2024 Jul 3;11:e55964. doi: 10.2196/55964 (PMC11255529; doi:10.2196/55964)
Supplement: Multimedia Appendix 2 [file humanfactors_v11i1e55964_app2.docx]

Excluded papers in full text eligibility phase.

**No primary paper (n=9)**

1. Montoye, A. H. Use of accelerometry and machine learning to measure free-living physical activity and sedentary behavior
2. Agarwal, S.; Gupta, M.; Khandelwal, S.; Jain, P.; Aggarwal, A.; Singh, D.; Mishra, V. K.; FitMe: A Fitness Application for Accurate Pose Estimation Using Deep Learning. 2021 2nd International Conference on Secure Cyber Computing and Communications (ICSCCC) - Volume 0, Issue 0, pp. 232-237. DOI: 10.1109/ICSCCC51823.2021.9478168
3. Agarwal, V.; Sharma, K.; Rajpoot, A. K. AI based Yoga Trainer - Simplifying home yoga using mediapipe and video streaming. 2022 3rd International Conference for Emerging Technology (INCET) - Volume 0, Issue 0, pp. 1-5
4. Singh, V.; Patade, A.; Pawar, G.; Hadsul, D. trAIner - An AI Fitness Coach Solution. 2022 IEEE 7th International conference for Convergence in Technology (I2CT) - Volume 0, Issue 0, pp. 1-4
5. Singh, N.; Singh, D. P.; Pant, B. A Comprehensive Study of Big Data Machine Learning Approaches and Challenges. 2017 International Conference on Next Generation Computing and Information Systems (ICNGCIS) - Volume 0, Issue 0, pp. 80-85
6. Schmitz, B.; Gatsios, D.; Pena-Gil, C.; Juanatey, J. R. G.; Prieto, D. C.; Tsakanikas, V.; Scharnagl, H.; Habibovic, M.; Schmidt, M.; Kleber, M. E.; De Bruijn, G. J.; Malberg, H.; Mooren, F.; Widdershoven, J.; Maerz, W.; Fotiadis, D.; Kop, W. J.; Bosch, J. Patient-centered cardiac rehabilitation by AI-powered lifestyle intervention - the timely approach. Atherosclerosis - Volume 355, Issue 0, pp. 251
7. Zarkogianni, K.; Athanasiou, M.; Mitsis, K.; Chatzidaki, E.; Polychronaki, N.; Perakis, K.; Vergeti, D.; Antonopoulou, D.; Papachristou, E.; Chioti, V.; Voutetakis, A.; Kalafatis, E.; Pervanidou, P.; Kanaka-Gantenbein, C.; Nikita, K. A comprehensive approach to empower self-management of health in childhood obesity based on gamification mechanisms and biofeedback. Diabetes Technology and Therapeutics - Volume 23, Issue 0, pp. A57-A58
8. Cartledge, S.; Rogerson, M.; Singh, T. K. R.; Huynh Huu, V.; Phung, D.; Gurrin, C.; Neil, C.; Ball, K.; Maddison, R. Seeing is believing: the feasibility and acceptability of using wearable cameras to enhance self-management of heart failure. European Heart Journal - Volume 40, Issue 0, pp. 96
9. Polce, E. M.; Kunze, K. N.; Fu, M. C.; Garrigues, G. E.; Forsythe, B.; Nicholson, G. P.; Cole, B. J.; Verma, N. N. Development of supervised machine learning algorithms for prediction of satisfaction at 2 years following total shoulder arthroplasty. J Shoulder Elbow Surg - Volume 30, Issue 6, pp. e290-e299

**Not artificial intelligence for increasing physical activity (n=33)**

1. Florian, Mueller; Damon, Young. 10 Lenses to Design Sports-HCI. BOOK
2. Gonçalo, Marques; Devin, Sherry; David, Pereira; Hammad, Fozi. Elevating Game Experiences with Unreal Engine 5: Bring your game ideas to life using the new Unreal Engine 5 and C++. BOOK
3. Konrad, Banachewicz; Luca, Massaron; Anthony, Goldbloom. The Kaggle Book: Data analysis and machine learning for competitive data science. BOOK
4. Lauren, Mullennex; Nate, Bachmeier; Jay, Rao. Computer Vision on AWS: Build and deploy real-world CV solutions with Amazon Rekognition, Lookout for Vision, and SageMaker. BOOK
5. Nguyen, Olivier. Population-level Indicators of Physical Activity, Sedentary Behaviour and Sleep in Canada based on Twitter
6. Noemí, Ferrera; Joe, Colantonio. How to Test a Time Machine: A practical guide to test architecture and automation. BOOK
7. Phil, Bramwell. Windows and Linux Penetration Testing from Scratch: Harness the power of pen testing with Kali Linux for unbeatable hard-hitting results. BOOK
8. Sumit, Gupta; Sylvester, Pinto; Shweta, Sankhe-Savale; Gillet, J. C.; Kenneth Michael, Cherven. The Tableau Workshop: A practical guide to the art of data visualization with Tableau. BOOK
9. Zhang, Shaoyan; Rowlands, Alex V; Murray, Peter; Hurst, Tina L. Physical activity classification using the GENEA wrist-worn accelerometer
10. Jang, K. J.; Ryoo, J.; Telhan, O.; Mangharam, R. Cloud Mat: Context-Aware Personalization of Fitness Content. 2015 IEEE International Conference on Services Computing - Volume 0, Issue 0, pp. 301-308
11. Jung, H. T.; Park, J.; Jeong, J.; Ryu, T.; Kim, Y.; Lee, S. I. A wearable monitoring system for at-home stroke rehabilitation exercises: A preliminary study. 2018 IEEE EMBS International Conference on Biomedical & Health Informatics (BHI) - Volume 0, Issue 0, pp. 13-16
12. Huang, S.; Wang, D.; Zhao, R.; Zhang, Q. Wiga: A WiFi-Based Contactless Activity Sequence Recognition System Based on Deep Learning. 2019 15th International Conference on Mobile Ad-Hoc and Sensor Networks (MSN) - Volume 0, Issue 0, pp. 69-74
13. Heiyanthuduwa, T. A.; Amarapala, K. W. Nikini Umasha; Gunathilaka, K. D. Vinura Budara; Ravindu, K. S.; Wickramarathne, J.; Kasthurirathna, D.. VirtualPT: Virtual Reality based Home Care Physiotherapy Rehabilitation for Elderly. 2020 2nd International Conference on Advancements in Computing (ICAC) - Volume 1, Issue 0, pp. 311-316
14. Kim, S.; Seo, D.; Lee, S.; Kim, Y.; Kang, H. W.; Choi, Y. S.; Jung, J. W. Real-Time Motion Feedback System based on Smart Mirror Vision. 2020 Joint 11th International Conference on Soft Computing and Intelligent Systems and 21st International Symposium on Advanced Intelligent Systems (SCIS-ISIS) - Volume 0, Issue 0, pp. 1-4
15. Chinchilla, S.; Salazar, J.; Hirata, Y. Tempo Synchronization of Physical Activities with a Mixed-Reality Human-Machine-Interface. 2021 IEEE International Conference on Robotics and Biomimetics (ROBIO) - Volume 0, Issue 0, pp. 364-371
16. Fieraru, M.; Zanfir, M.; Pirlea, S. C.; Olaru, V.; Sminchisescu, C. AIFit: Automatic 3D Human-Interpretable Feedback Models for Fitness Training. 2021 IEEE/CVF Conference on Computer Vision and Pattern Recognition (CVPR) - Volume 0, Issue 0, pp. 9914-9923
17. Sanz-De La Garza, M.; Villacorte, M.; Rodas, J. A.; Pi, R.; Redondo, J.; Gomez, J.; Zabala, I. SPODHA: Sport and health digital universe. European Journal of Preventive Cardiology - Volume 25, Issue 2, pp. S101
18. Gupta, A.; Gupta, H. P. YogaHelp: Leveraging Motion Sensors for Learning Correct Execution of Yoga With Feedback. IEEE Transactions on Artificial Intelligence - Volume 2, Issue 4, pp. 362-371
19. Sankaran, S.; Dendale, P.; Coninx, K. Evaluating the Impact of the HeartHab App on Motivation, Physical Activity, Quality of Life, and Risk Factors of Coronary Artery Disease Patients: Multidisciplinary Crossover Study. JMIR Mhealth Uhealth - Volume 7, Issue 4, pp. e10874
20. Armstrong, M.; Thomas, C. Incorporating virtual reality into a physical exercise programme for patients with parkinson's disease in an outpatient palliative care setting. Palliative Care and Social Practice - Volume 15, Issue 0, pp. 24
21. Wang, Z.; Shibai, K.; Kiryu, T. An Internet-based cycle ergometer system by using distributed computing. 4th International IEEE EMBS Special Topic Conference on Information Technology Applications in Biomedicine, 2003. - Volume 0, Issue 0, pp. 82-85
22. Schoeppe, S.; Duncan, M. J.; Plotnikoff, R. C.; Mummery, W. K.; Rebar, A.; Alley, S.; To, Q.; Short, C. E.; Vandelanotte, C. Acceptability, usefulness, and satisfaction with a web-based video-tailored physical activity intervention: The TaylorActive randomized controlled trial. J Sport Health Sci - Volume 11, Issue 2, pp. 133-144
23. Dias, S. B.; Oikonomidis, Y.; Diniz, J. A.; Baptista, F.; Carnide, F.; Bensenousi, A.; Botana, J. M.; Tsatsou, D.; Stefanidis, K.; Gymnopoulos, L.; Dimitropoulos, K.; Daras, P.; Argiriou, A.; Rouskas, K.; Wilson-Barnes, S.; Hart, K.; Merry, N.; Russell, D.; Konstantinova, J.; Lalama, E.; Pfeiffer, A.; Kokkinopoulou, A.; Hassapidou, M.; Pagkalos, I.; Patra, E.; Buys, R.; Cornelissen, V.; Batista, A.; Cobello, S.; Milli, E.; Vagnozzi, C.; Bryant, S.; Maas, S.; Bacelar, P.; Gravina, S.; Vlaskalin, J.; Brkic, B.; Telo, G.; Mantovani, E.; Gkotsopoulou, O.; Iakovakis, D.; Hadjidimitriou, S.; Charisis, V.; Hadjileontiadis, L. J. Users' Perspective on the AI-Based Smartphone PROTEIN App for Personalized Nutrition and Healthy Living: A Modified Technology Acceptance Model (mTAM) Approach. Front Nutr - Volume 9, Issue 0, pp. 898031
24. Seregni, A.; Tropea, P.; Re, R.; Biscaro, V.; Caprino, M.; Judica, E.; Corbo, M. Virtual coaching system for continuity of care and rehabilitation in patients with stroke. Results of the pilot study in the home scenario. Gait and Posture - Volume 97, Issue 0, pp. 29-30
25. Jain, D.; Bobrovsky, H.; Peters, C.; Cheng, A.; Hunt, D.; Decker, G.; Huber, S.; Goodwin, M. Validation of a mobile AI-based motion correction technology for physical therapy in treating patients with chronic low back pain-A pilot study. Global Spine Journal - Volume 12, Issue 3, pp. 39S-40S
26. McConnell, M. V.; Shcherbina, A.; Pavlovic, A.; Homburger, J. R.; Goldfeder, R. L.; Waggot, D.; Cho, M. K.; Rosenberger, M. E.; Haskell, W. L.; Myers, J.; Champagne, M. A.; Mignot, E.; Landray, M.; Tarassenko, L.; Harrington, R. A.; Yeung, A. C.; Ashley, E. A. Feasibility of Obtaining Measures of Lifestyle From a Smartphone App: The MyHeart Counts Cardiovascular Health Study. JAMA Cardiol –
27. Stein, N.; Brooks, K. A Fully Automated Conversational Artificial Intelligence for Weight Loss: Longitudinal Observational Study Among Overweight and Obese Adults. JMIR Diabetes - Volume 2, Issue 2, pp. e28
28. Vandelanotte, Corneel; Trost, Stewart; Hodgetts, Danya; Imam, Tasadduq; Rashid, Mamunur; To, Quyen G; Maher, Carol. Increasing physical activity using an just-in-time adaptive digital assistant supported by machine learning: A novel approach for hyper-personalised mHealth interventions. Journal of Biomedical Informatics - Volume 144, Issue 0, pp. 104435
29. Scott, B.; Richards, C.; Taitano, L.; Wu, T.; Zavareh, A.; Havandjian, L.; Raiszadeh, K.; Shahidi, B. THE INFLUENCE OF PATIENT-PROVIDER RISK EDUCATION ON REHABILITATION OUTCOMES IN INDIVIDUALS WITH CHRONIC BACK PAIN. Journal of Investigative Medicine - Volume 71, Issue 1, pp. 445
30. Zeng, M.; Nguyen, L. T.; Yu, B.; Mengshoel, O. J.; Zhu, J.; Wu, P.; Zhang, J. Convolutional Neural Networks for human activity recognition using mobile sensors. 6th International Conference on Mobile Computing, Applications and Services - Volume 0, Issue 0, pp. 197-205
31. Yin-Jun, C.; Yen-Chu, H. Using real-time acceleration data for exercise movement training with a decision tree approach. 2009 International Conference on Machine Learning and Cybernetics - Volume 5, Issue 0, pp. 3005-3010
32. Ranasinghe, I; Yuan, C; Dantu, R; Albert, M.V. A collaborative and adaptative feedback system for physical exercises. 2021. DOI: 10.1109/CIC52973.2021.00012
33. Cai, Z.; Fernando, O.N.N.; Ong, J.Y. PoseBuddy: Pose estimation workout mobile application. 2022. DOI: 10.1109/CW55638.2022.00034

**No access to full text (n=2)**

1. M. Habibovic, E. Douma, D. Gatsios, J. R. Gonzalez-Juanatey, C. Pena-Gil, J. Widdershoven, et al. A patient-centered intervention platform to support health behaviour change and the continuum of care in patients with coronary artery disease: The timely randomized controlled trials. Psychosomatic Medicine 2023 Vol. 85 Issue 4 Pages A53
2. K. Murphy, C. Davis, R. Curtis and C. Maher. Delivery of a 3-month Mediterranean diet and physical activity lifestyle intervention via artificial-intelligence chatbot, can achieve behaviour change: MedLiPal pilot-study. Proceedings of the Nutrition Society 2020 Vol. 79.
